# Investigation of Calcination of *Sepia officinalis* Cuttlefish Bone for Reinforcement of Polyvinyl Alcohol Added Nano-Size Montmorillonite

**DOI:** 10.3390/polym14061089

**Published:** 2022-03-09

**Authors:** Jia-Yi Thum, Lee Tin Sin, Soo-Tueen Bee, Jun-Ven Lim, Soo-Ling Bee

**Affiliations:** 1 Department of Chemical Engineering, Lee Kong Chian Faculty of Engineering and Science, Universiti Tunku Abdul Rahman, Jalan Sungai Long, Bandar Sungai Long, Kajang 43000, Malaysia; thumjiayi@1utar.edu.my; 2 Department of Mechanical and Material Engineering, Lee Kong Chian Faculty of Engineering and Science, Universiti Tunku Abdul Rahman, Jalan Sungai Long, Bandar Sungai Long, Kajang 43000, Malaysia; junvenlim@1utar.my; 3 School of Materials and Mineral Resources Engineering, Engineering Campus, Universiti Sains Malaysia, Nibong Tebal 14300, Malaysia; soolingbee2212@gmail.com

**Keywords:** biopolymers, polyvinyl alcohol, montmorillonite

## Abstract

This study aims to investigate the effects on calcination of *Sepia officinalis* cuttlefish bone (cuttlebone) to enhance reinforcement of polyvinyl alcohol (PVOH) added with nano-size montmorillonite (MMT) blends as potential bio-compatible materials. The polyvinyl alcohol-cuttlebone-montmorillonite nanocomposites were prepared using the solution casting method. Calcined cuttlebone particles were added to the PVOH matrix at different amount of 2 and 5 parts per hundred resin (phr) along with MMT ranging from 1 to 3 phr. Results showed that the tensile strength of cuttlebone-added PVOH-MMT composites at fixed 1 phr MMT was observed to be marginally lower when the cuttlebone increased from 2 phr to 5 phr due to the poor distribution of agglomerated particles. Nevertheless, at higher loading level of MMT, it was found that the addition of cuttlebone at 5 phr exhibited a reinforcing effect in PVOH-MMT blends. This is consistent with the scanning electron microscopy observation, where dispersion of a higher amount of cuttlebone in PVOH-MMT blends was observed to be more homogeneous than a lower amount of cuttlebone. Moreover, based on the X-ray diffraction analysis, the addition of cuttlebone significantly enhanced the intercalation effect of MMT particles in the PVOH matrix. Furthermore, the observation from infrared spectroscopy shows the amount of hydroxyl group for all composites reduced gradually with the increasing amount of cuttlebone. The addition of cuttlebone showed a “red shift” effect, indicating the formation of hydrogen bonds induced by cuttlebone. Lastly, lower enthalpy of melting was detected in relation to higher loading level of cuttlebone embedded in PVOH-MMT blends through differential scanning calorimetry. In conclusion, the blending of cuttlebone in PVOH-MMT is favorable to obtain better properties of composites.

## 1. Introduction

Nowadays, most plastic materials used are generally derived from petroleum or natural gas. These polymers are non-degradable and tend to accumulate in landfills after being used. Biodegradable polymers have gained tremendous attention from researchers over the past decades. Many studies have been carried out to develop environmentally-friendly materials [1]. Biodegradable polymers, which are also known as biopolymers, are defined as polymers that can be decomposed in the presence of enzymes or microorganisms under aerobic or anaerobic conditions. Biopolymers possess high versatility, microbiological degradability, and a diversity of applications, especially in the biomedical field [2]. Polyvinyl alcohol (PVOH) is one example of a biodegradable thermoplastic polymer. PVOH is produced from polyvinyl acetate through hydrolysis so that hydroxyl groups can be introduced on the main chains. PVOH has the characteristics of high water solubility and biocompatibility. It is also non-toxic and odorless. PVOH can form a highly orientated crystal structure, which is commonly applied in textile sizing, paper coating, and food packaging [3]. However, PVOH has the disadvantages of insufficient strength and low thermal stability [4]. In order to produce high performance and affordable polymeric materials, the thermal and mechanical properties and other functions of PVOH could be improved by incorporating biopolymers with other fillers [5].

Recently, polymer composites have been developed by dispersing foreign particles into polymer matrices. For example, natural fillers have been introduced to biopolymers to minimize the production cost. Natural fillers can be found from industrial or agricultural waste, such as fish bones, chicken bones, and eggshell, which can be incorporated in the polymer matrix [6,7,8]. The reason natural fillers are selected is due to their low density, high availability, renewability, and low energy consumption compared to synthetic fillers. They can also enhance the biodegradability of the composite materials. Hence, the polymer matrix is then hybridized with reinforcing filler to balance out the mechanical properties of composites. Montmorillonite and calcium carbonate are common reinforcing fillers [9].

In this research, montmorillonite (MMT) and calcined cuttlebone were used as potential reinforcing materials for a PVOH polymer. The selection of calcine cuttlebone was indeed because of its content of hydroxyapatite, which shares a similar composition to clam shell, chicken bones, fish bones, etc., which can be used to replace the high-cost synthetically-produced hydroxyapatite [6,7,8] used for orthopedic self-healing applications. On the other hand, owing to its high aspect ratio and large volume fraction of clay, merely adding small amounts of MMT can improve mechanical and tensile strength of nanocomposites to be better than traditional composites. MMT has the characteristics of thermal stability, inertness, and large surface area for interaction with the surrounding matrix [10]. Moreover, due to polymer intercalation, Young’s modulus and percentage of elongation of the nanocomposites will be higher than pure PVOH polymer [11]. MMT has hydrophilic characteristics due to the presence of sodium cations between its interlayers. It is miscible with a water-soluble hydrophilic polymer such as PVOH. Thus, a homogeneous dispersion in a polymer matrix can be formed in this polymer-clay nanocomposite [12]. In order to maintain the biological characteristic of the composite materials, the calcined cuttlebones were added to the composites as well. Such calcined cuttlebone is turned into hydroxyapatite, which possesses good characteristics of biocompatibility. However, MMT is cheap but often impure. Cuttlebone is also limited in supply and has reduced mechanical properties. Although there are some disadvantages of each raw material for nanocomposites, incorporation of reinforcing filler such as MMT can help improve the mechanical properties. Additionally, the composite material is suitable for application of biomaterials such as tissue and bone scaffolding materials [13]. It is also worth mentioning that the development of such materials meets the sustainability development goal (SDG) 12 of the United Nations, namely: Responsible Consumption and Production.

There are also previous studies within the research of incorporating fillers into polymers to create polymer composites with better properties. One of the literature reviews revealed that intercalated and exfoliated MMT can coexist in PVOH as PVOH/MMT blends based on X-ray diffraction analysis (XRD) and scanning electron microscopy (SEM) analysis [14,15,16,17]. Moreover, polyvinyl alcohol/surface-free single walled carbon nanotube (SF-SWCNT) nanocomposites were also able to be synthesized. The incorporation of SF-SWCNT can help in improving the mechanical strength and direct current (DC) electrical conductivity of nanocomposites [16]. Additionally, mechanical properties of polyvinyl alcohol nanocomposites incorporated with carbon nanotubes (CNTs) and MMT were also investigated. When the CNT amount was increased from 0.5 part per hundred resin (phr) to 1 phr, tensile strength and Young’s modulus of all PVOH-MMT blends increased. Fourier transform infrared (FTIR) results showed weak hydrogen bonding of all PVOH-MMT blends when low CNT (≤1 phr) was added. Low amounts of MMT particles still have the ability to exfoliate in the PVOH matrix. Low MMT amounts can improve the interaction effect between the PVOH matrix and CNT particles, whereas low CNT amounts improve mechanical and physical properties of pure PVOH and PVOH-MMT blends [14]. In addition, polyvinyl alcohol has been incorporated with eggshell powder using the method of solution blending and casting. The FTIR and SEM results have shown that the eggshell particles are well dispersed in the PVOH matrix due to hydrogen bonding interactions, showing that eggshell powder is an excellent biological filler [18]. Furthermore, acrylamide hydrogels have been modified with the addition of montmorillonite and kaolinite clay. The results showed that montmorillonite is more effective than kaolinite in the structure of hydrogels, due to its higher swelling ratio. Increases in basal spacing exhibited that the monomer had inserted into the interlayer of the clay [19]. This study was further extended with another clay, illite, and then compared with these three clays (montmorillonite, kaolinite, illite). Montmorillonite interacted strongly with the water and was still the best clay to be applied in the hydrogels [20].

## 2. Experimental

### 2.1. Materials and Formulation

The PVOH that was used in this experiment is a fully hydrolyzed PVOH with the grade of Sekisui Selvol^TM^ PVOH 103 (4% solution viscosity at 20 °C 4.00 cP and hydrolysis 98.4 mole%) that was manufactured by Sekisui Specialty Chemicals America, LLC. (Dallas, TX, USA). In this study, PVOH was used as the polymer base. The MMT used, which has a content of 0.5–5 wt% aminopropyltriethoxysilane and 15–35 wt% octadecylamine, was purchased from Sigma-Aldrich (M) Sdn. Bhd. (Petaling Jaya, Malaysia). MMT was used as the primary reinforcing filler in this study. Moreover, the cuttlefish (*Sepia officinalis*) bones (cuttlebone) were collected from fisherman at Pulau Ketam, Port Klang, Selangor, Malaysia. The purities of the PVOH and MMT were >95% and >96%, respectively. Since cuttlefish bone was naturally obtained, the purity of the cuttlefish bone was unidentified.

### 2.2. Preparation of Samples

#### 2.2.1. Preparation of Calcined Cuttlebone

The cuttlebone waste collected from the fisherman was washed thoroughly using tap water to remove dirt and residues. After that, the cleaned cuttlebones were dried under sunlight for three days. The dried cuttlebone was stored in a sealed bag until the calcination step. Before the calcination process, the dried cuttlebones were grinded into smaller particles using multipurpose grinder. The calcination step was carried out in a furnace at a heating temperature of 900 °C for three hours. Again, the calcined cuttlebone was grinded into a finer powder form with a porcelain mortar. There were 6 samples prepared with variations of MMT and cuttlebone with a constant amount of PVOH, as shown in Table 1. The amount of PVOH used was fixed at 10 g as the basis for other additives. Additionally, the amount of other additives to be added were expressed in part per hundred resin (phr) [2,4]. The montmorillonite was varied at 1 phr, 2 phr, and 3 phr, whereas the cuttlebone varied between 2 phr and 5 phr.

#### 2.2.2. Sample Preparation

The solution cast samples of PVOH-calcined cuttlebone-MMT were prepared. Firstly, PVOH and MMT were mixed and dissolved in 300 mL of distilled water at 97 ± 2 °C for 30 min using a water bath until all the PVOH dissolved. The amount of MMT was varied at 1 phr, 2 phr and 3 phr of PVOH. A stirrer was used to stir the mixtures for 1 h at room temperature. At this moment, suspension solution was formed. As the PVOH completely dissolved in distilled water, an additional 100 mL of distilled water was added together with the cuttlebone. Calcined cuttlebone was then added with the amounts of 2 phr and 5 phr. The PVOH-calcined cuttlebone-MMT blend was heated again in the water bath at the similar temperature, namely 97 ± 2 °C, for 1 h. The mixture was cast in the form of film in Petri dishes and dried in an oven at about 65 °C for about 48 h. The samples were sealed in sealed bags and stored at room temperature of 25 °C with 65% relative humidity.

### 2.3. Characterization Techniques

#### 2.3.1. X-ray Diffraction (XRD) Tests

X-ray diffraction analysis (XRD) tests were conducted to determine the dispersion state of MMT and calcined cuttlebone particles in the samples using a Shimadzu XRD 6000 X-ray diffractometer (XRD). The XRD spectra of all samples were recorded with the diffractometer using a Cu-Kα radiation generator (*λ* = 1.542 Å) at a scan rate of 1.2° min^−1^. The operation current and acceleration voltage of the Cu-Kα radiation generator were set at 30 mA and 40 kV, respectively. The interlayer spacing (also known as d spacing), *d*, was calculated according to Bragg’s equation, as shown in Equation (1). The inter-chain separation, *R*, was determined by using Klug and Alexander equation, as shown in Equation (2).

(1) $$d=\frac{\lambda }{2\;\sin\theta }$$

(2) $$R=\frac{5\lambda }{8\sin\theta }$$

where *λ* is 1.542 Å, and *θ* is the Bragg angle in radians.

#### 2.3.2. Tensile Tests

Tensile properties were determined using a model 5848 Instron tensile microtester in accordance with ASTM D882 (applying a rectangular film with a thickness of less than 1 mm). The cast samples of composites were cut into standard rectangular shapes of 6 mm width and 60 mm in length according to ASTM D882. The thickness of each specimen was measured using measuring instrument before proceeding to the tensile testing. Tensile tests were conducted with the strain rate and cell load of 50 mm/min and 5 kN, respectively. The tensile strength and Young’s modulus of each sample formulation were recorded as an average of five specimens.

#### 2.3.3. Fourier Transform Infrared Spectroscopy (FTIR) Analysis

Fourier transform infrared (FTIR) analysis was conducted using a Nicolet model IS10 FTIR spectrometer to determine the presence of specific chemical groups. The samples of composites were directly placed on the diamond crystal in the center of the ATR plate. The IR beam with 0.1 mm diameter of the FTIR instrument was focused at the center of the diamond. Pressure was applied to the sample in order to scan it by lowering the press of the ATR. Then, the dial of the ATR was turned until the click was heard. The samples were scanned under the region of 4000 cm^−1^ to 400 cm^−1^.

#### 2.3.4. Scanning Electron Microscopy Analysis (SEM)

The fractured surface morphologies of the composites were observed by using a Hitachi S3400N SEM machine. The applied electron beam voltage of the SEM machine was set at 15 kV. Firstly, the fractured surfaces of all composites were cut into smaller portions. The cut samples were then mounted onto specimen stubs using double sided carbon tape with the fractured surfaces of the cut samples placed facing up. The mounted samples were then coated with a layer of gold and palladium using an EMITECH SC7620 sputter coater. The coated samples on stubs were loaded into the SEM chamber and scanned with an electron beam voltage of 30 kV. The SEM micrographs were recorded at the magnifications of 1000×, 3000×, and 10,000×.

#### 2.3.5. Differential Scanning Calorimetry (DSC)

DSC analysis was performed using a Mettler Toledo DSC823. The samples with weights of 1–10 mg were determined and loaded into crucibles. Scanning was carried out from 30 °C to 200 °C at a scanning rate of 10 °C/min under dry nitrogen of nearly 100% purity at a purge rate of 20 mL/min. The DSC thermograms were used to obtain the onset and endpoint melting temperatures for the samples.

## 3. Results and Discussion

### 3.1. Tensile Test Analysis

**Figure 1:**
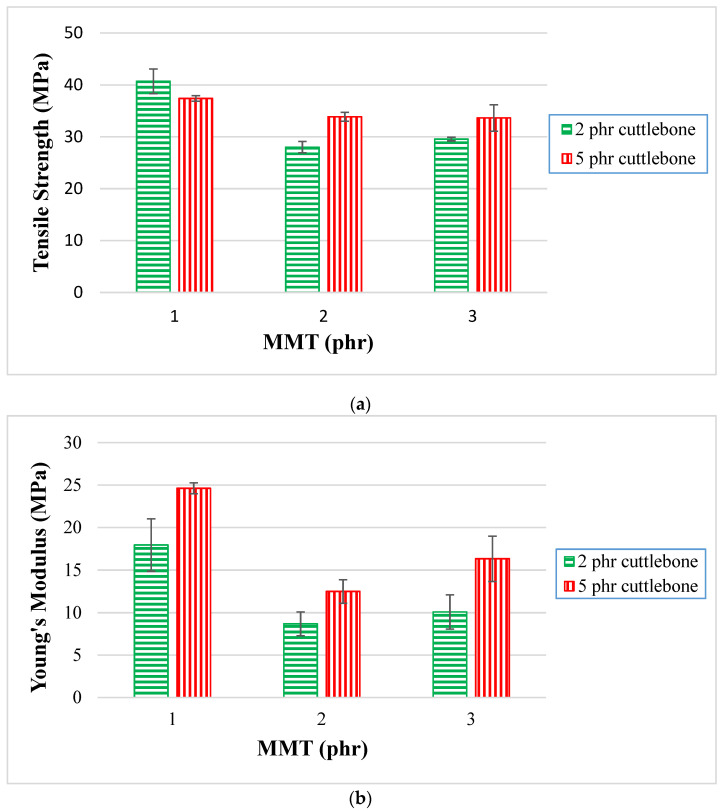

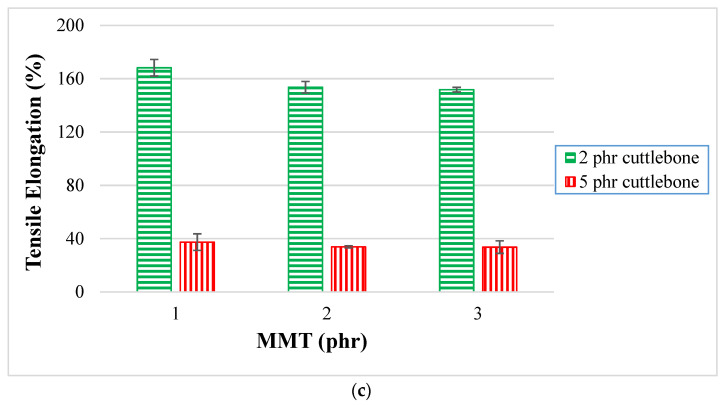
(**a**) Tensile strength, (**b**) Young’s modulus, and (**c**) tensile elongation (%) for PVOH-calcined cuttlebone-MMT nanocomposites.

By referring to Figure 1a, various loading levels of MMT ranging from 1 phr to 3 phr were added into PVOH with fixed amounts of 2 phr or 5 phr of calcined cuttlebone. When different amounts of MMT were incorporated with PVOH at the fixed 2 phr calcined cuttlebone loading level, the highest tensile strength was observed with a value of 41 MPa when 1 phr of MMT was added. The tensile strength then decreased from 41 MPa to 28 MPa, and further increased again to 29 MPa when the MMT was gradually increased to 2 and 3 phr respectively. The reduced tensile strength was attributed to the non-homogeneous distribution of MMT on the PVOH matrix. Agglomeration of MMT particles occurred, causing stress concentration on the polymer matrix when subjected to extension. The PVOH matrix also had a poor intercalation effect in MMT galleries. Nevertheless, tensile strength of PVOH-calcined cuttlebone composites increased when the amount of MMT was added to 3 phr. This is because PVOH has an extensive amount of hydroxyl functional groups that would interact excellently with the inherent hydrophilic characteristics of MMT. Hydrogen bonding could be formed to bind MMT strongly with the PVOH matrix. A high amount of MMT would act as a lubricating agent to slide between PVOH-cuttlebone blends. The good intercalation of MMT promoted the effective transfer of extension loading of MMT to silicates.

On the other hand, when the amount of calcined cuttlebone was increased to a fixed 5 phr loading level, it was found that the tensile strength of PVOH-calcined cuttlebone-MMT nanocomposites decreased gradually with the increase in the amount of MMT incorporated. This result was observed to be similar to that of the composites added with varied MMT loading levels at fixed 2 phr of calcined cuttlebone. This is due to the uneven dispersion of MMT agglomerates, causing inferior effects to the PVOH matrix. The phase separation ruined the continuity of the PVOH matrix and further weakened the mechanical performance of the nanocomposites.

Moreover, Figure 1a also shows nanocomposites with various amount of calcined cuttlebone incorporated with PVOH with fixed 1 phr, 2 phr, and 3 phr of MMT. At the fixed low amount of 1 phr MMT, as the loading level of calcined cuttlebone increased, the tensile strength decreased. This indicates that calcined cuttlebone was incompatible with MMT and PVOH due to the agglomeration of calcined cuttlebone particles. The calcined cuttlebone tended to form stronger hydrogen bonding within themselves rather than within the PVOH matrix. Hence, less energy was required to break the weak intermolecular force of PVOH, causing the reduction of tensile strength overall. The cuttlebone was considered to give less reinforcing effect to the PVOH matrix at low loading levels of MMT.

However, when the loading levels of MMT were fixed at 2 phr and 3 phr, the increasing calcined cuttlebone caused an increase in tensile strength. Since the fillers and PVOH contain hydroxyl groups, calcined cuttlebone formed strong hydrogen bonds with the PVOH matrix and MMT; this good compatibility between the materials within the composite resulted in the composite being able to bear more stress load. This is in line with the observation of Soltani et al. [21], who reported that cuttlebone have a nucleation role that can change the crystallographic orientation of nucleation and control its location. This resulted in uniform stress distribution and less voids with each other. The enhanced tensile strength was achieved, as the agglomeration of cuttlebone could be counterbalanced by the addition of high amounts of MMT.

Figure 1b illustrates Young’s modulus of various PVOH-cuttlebone-MMT nanocomposites, showing similar results with the observation for the tensile strength test. When the amount of cuttlebone was fixed at 2 phr with increasing loading levels of MMT, there was a drop in Young’s modulus by 50%, before moving up by 14%. Young’s modulus declined significantly due to the agglomeration of MMT. This is because there was poor adhesion between the MMT and PVOH-calcined cuttlebone blends, as the particles competed with each other to form hydrogen bonds with the polymer matrix. A weak interface zone was then generated, causing disintegration of the bonds. Furthermore, Young’s modulus increased a little when the amount of MMT was up to 3 phr. This could be explained by MMT forming firm hydrogen bonds with the PVOH matrix, which helps in restricting polymer chain motions. High stress and low strain resulted in a higher modulus value, causing the composites to possess a high resistance against movement. When the amount of cuttlebone was fixed at 5 phr, the Young’s modulus of PVOH-cuttlebone-MMT nanocomposites fell gradually from 25 MPa to 13 MPa and further increased up to 16 MPa, with increasing MMT. This is similar to the situation when the cuttlebone was added at the fixed amount of 2 phr. However, one of the more noticeable results is the highest Young’s modulus of the polymer nanocomposites, with the value of 25 MPa ± 0.64 for 1 phr of MMT and 5 phr cuttlebone incorporated with PVOH. It had the highest rigidity and stiffness, which is attributed to the good dispersion between the PVOH matrix, MMT, and cuttlebone fillers. Figure 1b also depicts various loading levels of cuttlebone; 2 phr and 5 phr were added into PVOH with fixed 1 phr, 2 phr, and 3 phr of MMT. The trend where the Young’s modulus of composites with 5 phr of cuttlebone was consistently higher than that of the composites with a loading level of 2 phr of cuttlebone was observed for all fixed loading levels of MMT. This showed that cuttlebone fillers provided significant effects on reinforcement for nanocomposites. There was strong intermolecular and intramolecular hydrogen bonding within nanocomposites. Owing to the properties of high rigidity and stiffness of cuttlebone, the cuttlebone particles interacted and dispersed homogeneously in the PVOH matrix, contributing to high Young’s modulus.

Moreover, Figure 1c presents tensile elongation at various loading levels of MMT, ranging from 1 phr to 3 phr added into PVOH with fixed 2 phr and 5 phr of cuttlebone. When the cuttlebone was fixed at 2 phr, the tensile elongation decreased gradually, which was caused by the agglomeration of the high loading level of MMT. Thus, it can be seen that MMT was unable to be distributed evenly in the PVOH matrix, eventually causing the region stress concentration to become more pronounced in the polymer matrix. An irregular matrix chain was also produced as the formed clusters MMT prevented the smooth flow of PVOH. The addition of MMT might increase the brittleness of polymer blends, contributing to the reduction of the ability to deform before fracture. This resulted in lower elongation at break, as the recrystallization was restricted during deformation. It could be observed that the highest tensile elongation was at 100 phr PVOH-2 phr calcined cuttlebone-1 phr MMT composite due to the high strain at its breaking point. The observation for fixed 5 phr calcined cuttlebone was similar with the previous observation when the calcined cuttlebone was fixed at 2 phr. This is shown when the percentage tensile elongation graph shows a downward trend for all fixed loading levels of MMT. Additionally, the tensile elongation for nanocomposites with loading level of 5 phr calcined cuttlebone is consistently lower that that with 2 phr of cuttlebone. This is because the increased amount of calcined cuttlebone would induce poor dispersion of cuttlebone particles on the PVOH matrix due to the aggregation of calcined cuttlebone particles. Additionally, more stress concentration region was formed along the calcined cuttlebone particles leading to the reduction of chain sliding ability. The reinforcing effect of calcined cuttlebone was insignificant, whereas the polymer ductility was reduced and caused the elongation to drop dramatically. Overall, only a small amount MMT and calcined cuttlebone fillers was required to exhibit a good reinforcing effect to the PVOH matrix, as elongation at break obviously exhibited the highest result at the nanocomposite incorporated with PVOH, 1 phr MMT, and 2 phr calcined cuttlebone. To test the tensile elongation of the polymer chain, the chain was pulled with a hook, thus extending the length of the PVOH chain and increasing the elongation of the polymer composites.

### 3.2. Differential Scanning Calorimetry

**Figure 2:**
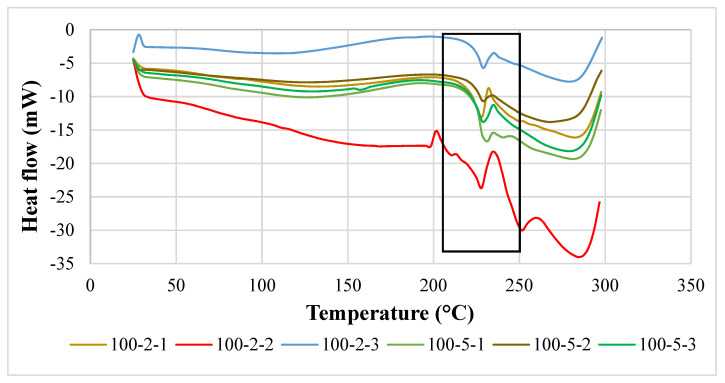
DSC thermogram of PVOH-calcined cuttlebone-MMT composites. Note: 100-2-1 denotes 100 phr PVOH added with 2 phr cuttlebone and 1 phr MMT.

**Figure 3:**
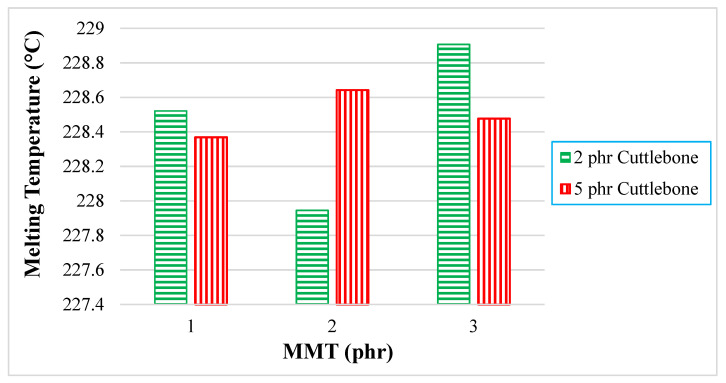
Effect of increasing MMT and calcined cuttlebone loading level on melting temperature.

A differential scanning calorimetry (DSC) thermogram of PVOH-cuttlebone-MMT is shown in Figure 2. An obvious endothermic peak could be observed at the region of 200 to 250 °C. Moreover, the extent of the melting state of the DSC is also a vital element to express the magnitude of intermolecular bonding in the blends. When there is large peak area or high enthalpy of melting, more thermal energy is needed to convert into kinetic energy to allow the polymer molecules to free out from the ordered crystalline structure. Figure 3 shows the thermogram of the composites. At fixed 2 phr calcined cuttlebone, when the amount of MMT was added from 1 phr to 2 phr, the melting temperature decreased. This is because MMT interrupted the interaction of calcined cuttlebone and polymer matrix, forming a larger number of particles with a higher size of irregularity, promoting the occurrence of agglomeration. When the chain arrangement of the polymer matrix was disrupted, this led to non-homogeneous dispersion of MMT on PVOH-cuttlebone polymer blends. On the other hand, when the amount of MMT increased from 2 phr to 3 phr, the melting temperature increased, and this indicated that MMT was acting as an enhancement phase to form secondary bonding. This indicated that there was good interaction of cuttlebone and MMT with the PVOH matrix towards forming a highly stable structure, as the previous section mentioned that cuttlebone and MMT had hydrogen bonds formed with the PVOH matrix. Moreover, the rigidity of the composite increased, and more heat energy was needed to vibrate the stronger hydrogen bonding. On the other hand, when the increment of cuttlebone loading level was increased to 5 phr with fixed 1 phr and 3 phr MMT loading levels, the melting temperature decreased. This was due to the agglomeration of cuttlebone, which resulted in uneven distribution of the composites. This condition could be explained with the interfacial adhesion being weakened, thus leading to formation of weaker hydrogen bonding of particles and PVOH. This caused irregular chain arrangement of the PVOH matrix that reduced the existing hydrogen bonding strength within the polymer matrix. This observation was indeed supported by the decreasing tensile strength results, as discussed in the earlier section. However, when the cuttlebone loading level increased to 5 phr and the amount of MMT was fixed at 2 phr, thermal stability was enhanced due to the increasing cuttlebone content. This was attributed to the high rigidity and strength of polymer nanocomposites, which was caused by hydrogen bond formed with the cuttlebone fillers, which inhibited the mobility of the chain, eventually effectively improving the melting temperature [22]. This result was in agreement with the FTIR observations, where the O-H stretching shifted to a lower wavenumber when there was an increase in calcined cuttlebone loading level. The wavenumber of O-H stretching also had a similar trend as the tensile strength results with increasing MMT amount. This result means that high concentrations of calcined cuttlebone and MMT can induce formation of strong PVOH-cuttlebone-MMT, although agglomeration might still occur. Overall, the superior effect of formation of a large number of hydrogen bonds might overshadow the inferior effect of agglomeration of calcined cuttlebone. This is seen when the addition of MMT compensated for the inferior effects of calcined cuttlebone in the blends [23].

**Figure 4:**
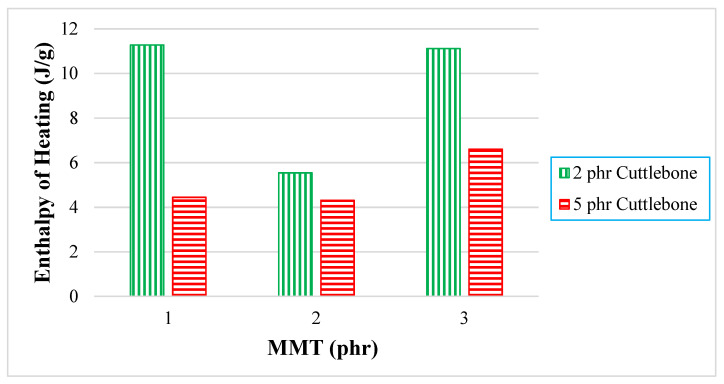
Effect of increasing MMT and calcined cuttlebone loading level on enthalpy of melting.

For quantitative analysis, the enthalpy of melting (∆H_M_) extracted from the data are tabulated and plotted in Figure 4. When there was an increment of MMT from 1 phr to 2 phr for all fixed loading levels of cuttlebone (2 and 5 phr), the enthalpy of melting decreased. This is because the interaction effect of MMT and polymer matrix was reduced, whereas when there was an increasing amount of MMT from 2 phr to 3 phr for fixed 2 and 5 phr loading levels of cuttlebone, the enthalpy of melting was shown to increase. This is because inorganic layers of MMT promoted a new crystal structure, leading to better thermal properties. The stiffening behavior of MMT also improved the crystallinity of PVOH-calcined cuttlebone-MMT nanocomposites. In fact, MMT tended to enhance the reinforcing effect through physical interaction, such as intercalation and exfoliation effects, to boost the mechanical strength of the composites [23]. The interaction of MMT and calcined cuttlebone became intense when blended with PVOH. The addition of MMT at higher amounts helped to recover the strong interaction in PVOH-calcined cuttlebone blends. Both reinforcing fillers were well dispersed in the PVOH matrix. Similarly, this was evidently shown when the tensile strength for PVOH blends with 3 phr MMT and 2 phr calcined cuttlebone were higher than the PVOH blends with 2 phr MMT and 2 phr cuttlebone. Moreover, according to Young’s modulus data, PVOH blends with 3 phr MMT and 2 phr cuttlebone had higher rigidity with an increment of 16.24% when compared with PVOH blends with 2 phr MMT and 2 phr cuttlebone.

Further examination indicated that when there was an increment of calcined cuttlebone from 2 phr to 5 phr incorporated for all fixed loading levels of MMT, it showed that the enthalpy of melting decreased. The reason that the addition of calcined cuttlebone in the PVOH caused significant reduction of enthalpy of heating was due to the weakening structure of cuttlebone, which ruined the intermolecular bonding beyond expectation. Indeed, the irregularity of cuttlebone caused this inferiority [23], limiting the formation of primary bonding. As a result, the crystallinity was suppressed by the introduction of calcined cuttlebone into the PVOH matrix. Table 1 shows the melting temperature, onset temperature, and end temperature for all PVOH-calcined cuttlebone-MMT composites. As noticed for the onset temperature for all samples, with the increment of calcined cuttlebone to the 5 phr loading level, it shifted to a higher temperature for each fixed amount of MMT. This is due to the fact that the addition of both MMT and calcined cuttlebone promoted thermal stability, as is usually found in outstanding blending materials.

### 3.3. Infrared Spectrometry Analysis

**Figure 5:**
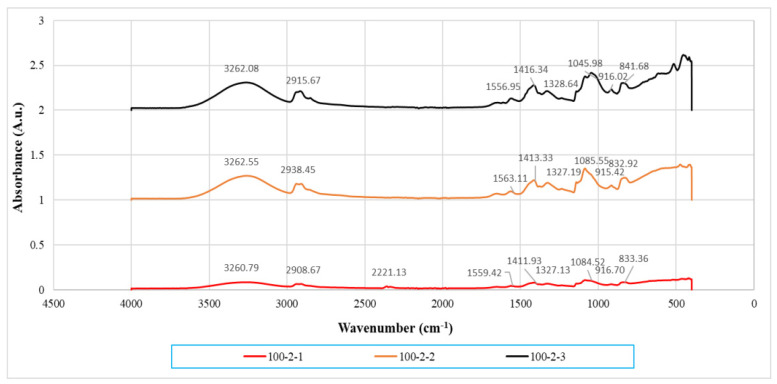
Infrared spectrum of PVOH-MMT nanocomposites added with 2 phr of calcined cuttlebone. Note: 100-2-1 denotes 100 phr PVOH added with 2 phr cuttlebone and 1 phr MMT.

**Figure 6:**
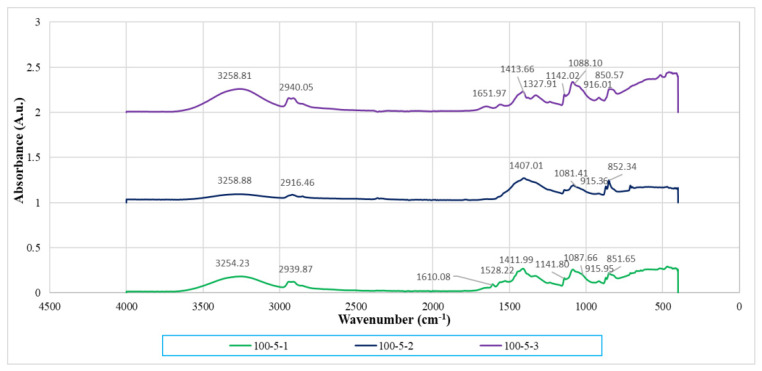
Infrared spectrum of PVOH-MMT nanocomposite added with 5 phr calcined cuttlebone. Note: 100-5-1 denotes 100 phr PVOH added with 5 phr cuttlebone and 1 phr MMT.

By referring to Figure 5 and Figure 6, the most important peak of 3200–3300 cm^−1^ represents stretching of the hydroxyl (-OH) functional group (referring to Table 2). This broad and significant peak was observed to identify the extent of the hydrogen bonding interaction of the composites. There were also several peaks visible, which were the C-H functional group in the region of 2908.67–2940.05 cm^−1^ and C-O stretching in the range of 1045.98–1088.10 cm^−1^. Hydroxyl bending was found at the wavenumber range of 1407.01–1416.34 cm^−1^. C-H bending could also be observed at the range of 1327.13–1328.64 cm^−1^. As reported by Lim [24], the pure PVOH has vibration of the hydroxyl group at around 3282.82 cm^−1^. Thus, when comparing with pristine PVOH, the shifting of band position of stretching vibration of hydroxyl groups of all the PVOH-calcined cuttlebone-MMT composites to a lower frequency could be observed. This was induced by a red shift effect of hydrogen bonding action, indicating the formation of strong hydrogen bonding between PVOH, MMT, and calcined cuttlebone [19]. The reason is that PVOH is hydrophilic, while calcined cuttlebone and MMT also have hydroxyl groups on the surface, promoting the hydrophilic nature of PVOH. Moreover, the interlayer gallery of MMT enabled the intercalation of polymer chains and cuttlebone to strengthen such interactions. As a result, internal hydrogen bonding between polymer chains in the PVOH matrix was weakened due to the good dispersion and interaction of MMT and cuttlebone in the PVOH matrix, hence producing synergistic effects [23].

At fixed 2 phr and 5 phr calcined cuttlebone, when the MMT loading level increased to 2 phr, the hydroxyl groups shifted to a higher wavenumber. This denotes the weakening of hydrogen bonding with the PVOH matrix. While it should be noted that the addition of calcined cuttlebone and MMT did not disrupt the presence of hydrogen bonding in the PVOH matrix, they tended to agglomerate together, causing poor dispersion of both fillers in the PVOH matrix. This observation was in agreement with the tensile strength results that increasing the amount of MMT could introduce agglomeration [25]. In other words, the aggregated MMT and cuttlebone particles significantly weakened the interaction effect between fillers and the PVOH matrix and thus promoted the hydrogen bonds within the PVOH matrix itself. However, there was a subsequent reduction of OH-stretching wavenumber of PVOH-cuttlebone-MMT composites when the amount of MMT reached 3 phr. This indicates the formation of more hydrogen bonds, inducing exfoliation and intercalation effects of MMT, which improved the tensile strength of the composites. Additionally, in order to further investigate the effects of varying calcined cuttlebone-added PVOH-MMT blends, when the calcined cuttlebone was increased to 5 phr in all of the fixed MMT amounts, the OH-stretching wavenumber of composites was observed to be lower when compared to 2 phr calcined cuttlebone. Such desirable interactions between calcined cuttlebone and PVOH-MMT blends were detected. Thus, it was believed that the enhancement of tensile strength was contributed by the addition of a low amount of hydrophilic MMT (1 phr), along with the strong secondary hydrogen bonding that was formed, which provided additional reinforcing interaction among components in the blends. The evidence for this could be shown as the wavenumber of the O-H functional group in PVOH composite with 1 phr MMT and 5 phr calcined cuttlebone was the lowest, which was 3254.23 cm^−1^.

Additionally, the wavenumber of C-H and C-O stretching was reduced when the amount of MMT increased to 2 phr then slightly increased at 3 phr MMT, at fixed loading level of 5 phr calcined cuttlebone. The reduction of wavenumber of C-H and C-O stretching indicated that the addition of MMT particles can enhance the strength of C-H and C-O bonding inside the PVOH matrix, which further strengthens the structure of PVOH. Thus, the addition of MMT significantly improved the mechanical properties of composites such as tensile strength. Moreover, higher energy is required to overcome the effect caused by the higher stiffness of molecular structure when there is shifting of the wavenumber to a high frequency.

### 3.4. X-ray Diffraction

**Figure 7:**
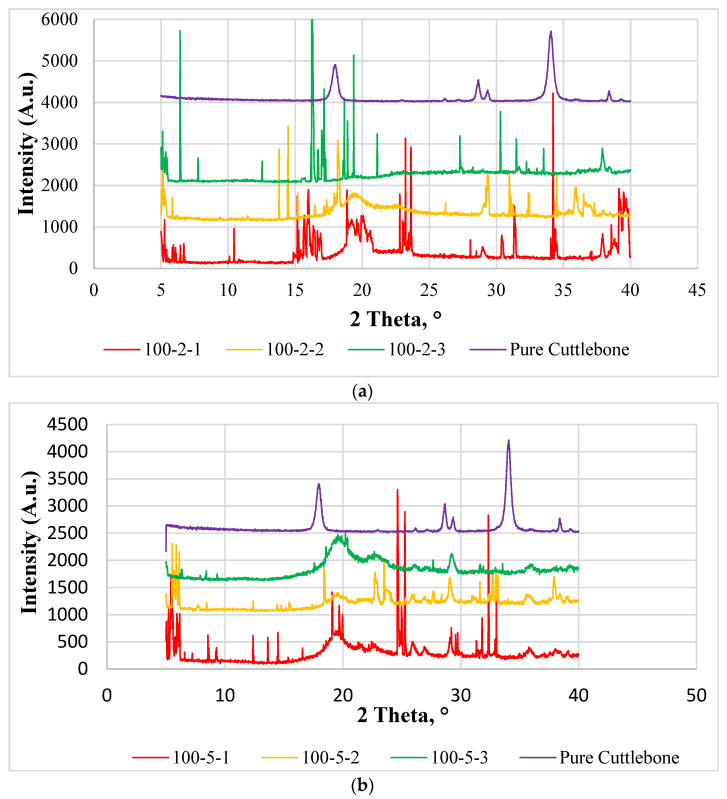
XRD curves (5° ≤ 2θ ≤ 40°) for (**a**) pure calcined cuttlebone and PVOH added with 2 phr of calcined cuttlebone and various loading levels of MMT, and (**b**) pure calcined cuttlebone and PVOH added with 5 phr of calcined cuttlebone and various loading levels of MMT. Note: 100-5-1 denotes 100 phr PVOH added with 5 phr cuttlebone and 1 phr MMT; others followed this notation as well.

Figure 7 shows the XRD patterns of varying calcined cuttlebone-added polyvinyl alcohol nanocomposites with fixed amounts of montmorillonite from 2θ = 5° to 2θ = 40°. Wei et al. [26] reported that the broad peak at 2θ = 19.4° assigned to (101) was caused by the diffraction of the crystal plane of PVOH, which was due to the intermolecular hydrogen bonding between PVOH chains. This XRD pattern demonstrated its semi-crystalline structure because it had high affinity to form hydrogen bonds. Overall, when the higher amount of calcined cuttlebone fillers were embedded into the PVOH matrix, they were well dispersed until the crystallite structure disappeared. As observed, the calcined cuttlebone had clear crystals which underwent random crystallization after addition into matrix. However, PVOH affected the highly ordered structure of calcined cuttlebone, causing disappearance of deflection peaks at 18°, 26°, and 28°. The molecules were changed due to phase separation of composites, leading to good interaction with PVOH chains. Furthermore, it can be observed that MMT had some effects on the interaction with the PVOH matrix due to the presence of MMT defection peaks. Poor dispersion of MMT in the PVOH matrix resulted in agglomeration of MMT particles when MMT was incorporated with the PVOH matrix. Hence, formation of crystallite structures was induced by organic MMT, eventually generating most of the sharp crystallite peaks on XRD curves.

Table 3 presents the crystallite size and d-spacing at 2θ = 19.4° for all PVOH-calcined cuttlebone-MMT composites. At fixed 2 phr calcined cuttlebone loading level, it can be observed that addition of MMT loading level could lower the intercalation effect of MMT in the PVOH matrix by referring to the decrease in d-spacing, which could be caused by the agglomeration of MMT particles. The crystallite size also decreased from 611.41 Å to 84.86 Å and then increased to 611.05 Å, as shown in Table 3. MMT particles were also separated from the PVOH-cuttlebone phase. The decrement of d-spacing increased the compactness on this deflection peak. The d-spacing of polymer composites was directly related to the tensile strength of composites. This implies that when the d-spacing decreased, the tensile strength of polymer composites decreased. However, based on Table 3, 100 phr PVOH-5 phr calcined cuttlebone-1 phr MMT and 100 phr PVOH-5 phr calcined cuttlebone-2 phr MMT had no signature peak at 19.4°, representing that the calcined cuttlebone and MMT interacted well with the PVOH matrix, producing random chains in the composites.

**Figure 8:**
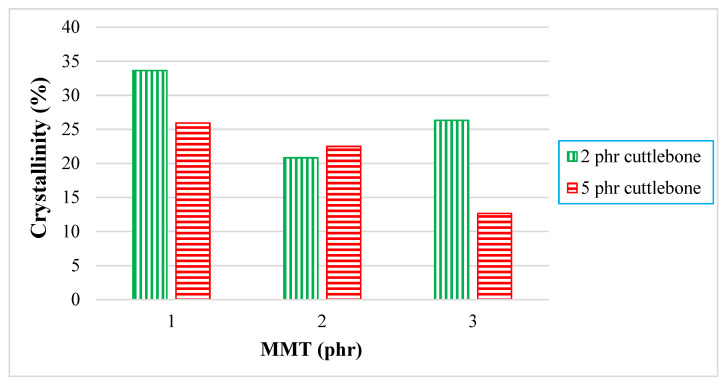
Crystallinity of PVOH-calcined cuttlebone-MMT composite.

Figure 7a displays XRD curves for PVOH blends incorporated with 2 phr cuttlebone and various amount of MMT, whereas Figure 7b illustrates XRD curves for PVOH blends incorporated with 5 phr cuttlebone and various amount of MMT. Additionally, the effect of various MMT and cuttlebone loading levels on crystallinity of PVOH nanocomposites is shown in Figure 8.

By referring to Figure 8, for 2 phr of cuttlebone loading, the crystallinity of composites was slightly decreased from 33.65% to 20.82%, and then increased to 26.33% when the various loading levels of MMT were added into the PVOH matrix. This decrement identified that the addition of MMT could slightly disrupt the arrangement of the crystal structure in the PVOH matrix. For example, it could rupture the orientation of the crystalline structure in the polymer matrix of PVOH composites. Additionally, there could be non-homogeneous distribution of MMT, which prevents the MMT particles from getting into the PVOH-cuttlebone blends. When the regularity of the matrix chain arrangement was reduced, this resulted in transformation of the structure from crystallite to amorphous and thus reduced the crystallinity. Moreover, the increased crystallinity indicated that the fillers exhibited good interaction with the polymer matrix, inducing the formation of more crystallite structure. This is because calcined cuttlebone and MMT have hydrophilic characteristics and contain formed hydroxyl groups. They tended to form new hydrogen bonds with the polymer matrix, inducing the formation of new crystallites, which resulted in a more orderly chain arrangement. These results were in agreement with the tensile strength test. Furthermore, when the loading level of cuttlebone increased, the crystallinity decreased. This is due to the agglomeration of cuttlebone in the PVOH matrix, causing the decrease of interfacial adhesion to occur. Thus, no new bond or crystalline structure would be formed. It could be observed that the mechanisms for addition of MMT and calcined cuttlebone on polymer blends are analogous, leading to similar trends for d-spacing as well as the tensile strength test.

### 3.5. Scanning Electron Microscope

**Figure 9:**
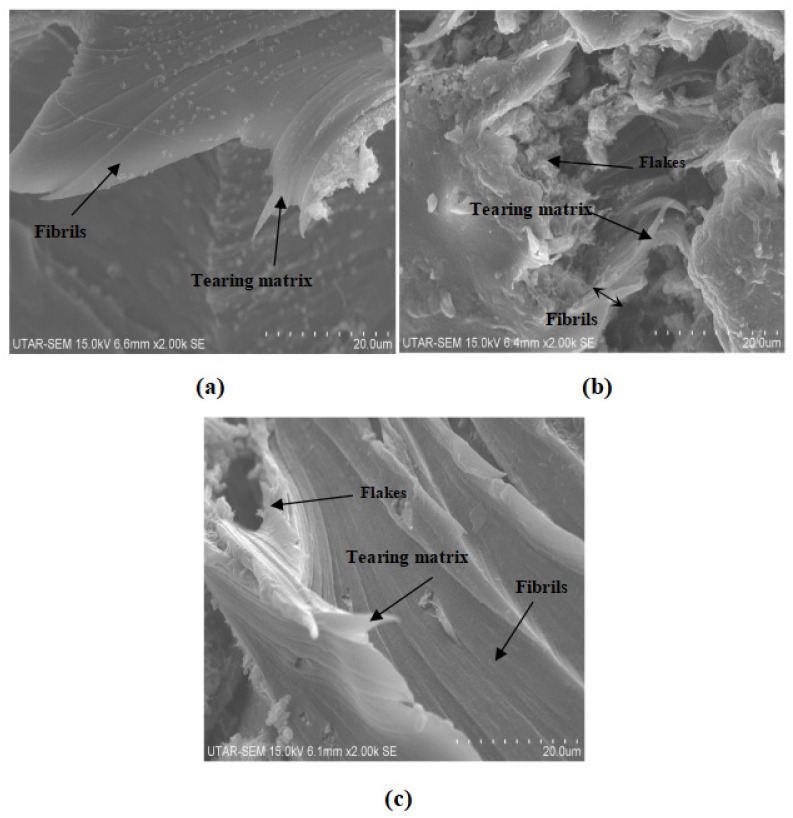
SEM micrograph of 2 phr calcined cuttlebone incorporated into PVOH matrix added with (**a**) 1 phr MMT, (**b**) 2 phr MMT, and (**c**) 3 phr MMT.

**Figure 10:**
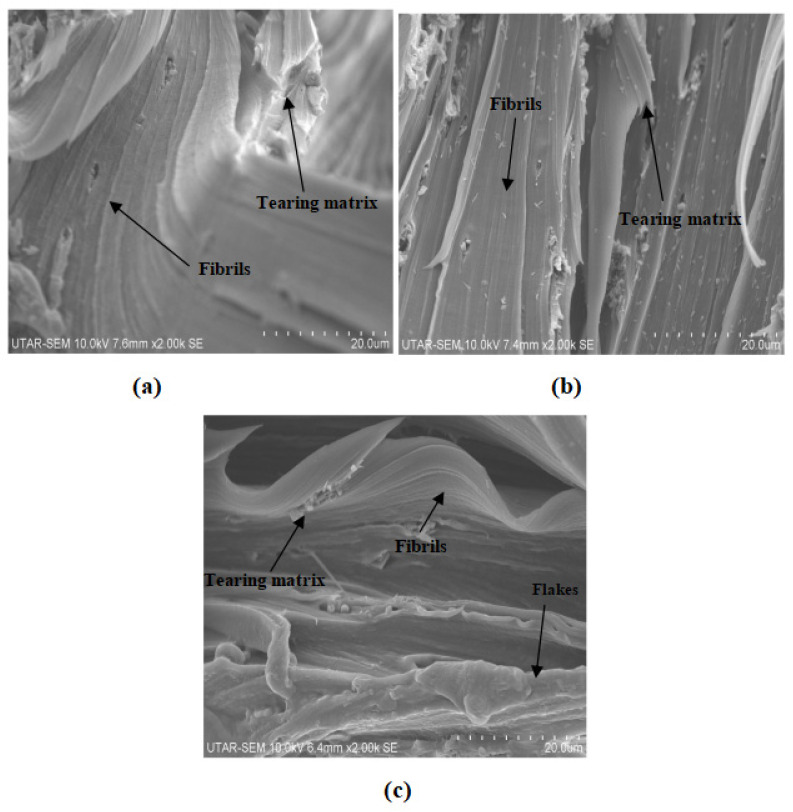
SEM of 5 phr calcined cuttlebone incorporated into the PVOH matrix added with (**a**) 1 phr MMT, (**b**) 2 phr MMT, and (**c**) 3 phr MMT.

Figure 9 and Figure 10 show the fracture surface morphologies of all calcined cuttlebone-added PVOH nanocomposites at various loading levels of MMT. For all the composites at fixed 2 phr calcined cuttlebone, there were formations of fibrils in the PVOH matrix due to the resistance ability of the matrix from being elongated. The tearing matrix appeared for all varied amounts of MMT in PVOH-calcined cuttlebone composites because of the discontinuity matrix form. In addition, when the MMT was at 1 phr, it exhibited the best matrix continuity, which indicated homogeneous distribution of MMT particles. It is suggested that the MMT formed hydrogen bonding with the PVOH matrix, which contributed to good interfacial adhesion of MMT and thus good dispersion within the PVOH matrix. The resistant tearing effect of the PVOH matrix was improved by extending the continuity of macromolecule chains. This caused the load to be transferred more effectively, enabling the matrix to elongate more when stress was applied. The blend was also able to break in an ordered direction throughout the matrix by producing long fibrils. This is further in agreement with tensile strength results, with the PVOH nanocomposite with 2 phr calcined cuttlebone and 1 phr MMT loading level representing the highest tensile strength and tensile elongation. Moreover, by referring to Figure 9b, when the amount of MMT increased to more than 1 phr, flake-like structures were present on the surface of the nanocomposites. Additionally, the flake-like structures were seen to be increased drastically with increasing MMT filler. This implies that increasing the MMT amount could promote the agglomeration effect of MMT in the PVOH matrix. Non-homogeneous distribution of MMT particles in PVOH caused by agglomerates also hindered mobility of slippage in the PVOH chain during crystallization. Furthermore, the agglomeration of MMT formed a stress concentration region, contributing to the ineffective transfer of load. Short fibrils are also observed in Figure 9b. This observation is further supported with the decrease in tensile strength and tensile elongation when the amount of MMT added to the composite increased, as shown in Figure 1a,c.

Moreover, less agglomerates can be seen in Figure 9c, confirming that the tensile strength for 3 phr MMT with PVOH-calcined cuttlebone blends was higher than 2 phr MMT with PVOH-calcined cuttlebone blends. There was only slight aggregation where the effect was insignificant. The superior properties overrode the inferior properties, contributing to the good continuity of macromolecule chains. At fixed 5 phr calcined cuttlebone, when the amount of MMT was at 1 phr, the continuity of the matrix was smooth, as seen in Figure 10a. However, when the amount of MMT increased to 2 phr and 3 phr, distinct characteristics on the composites could be observed, such as tearing matrix, flake-like structure, and fibrils exhibited on the surface of composites, as shown in Figure 10b,c. As a result, the structures of nanocomposites were slightly changed, especially for 3 phr MMT incorporated PVOH-cuttlebone blend (Figure 10c). The brittleness effect increased and caused the nanocomposites to break faster when high stress was applied. Therefore, they eventually provided low tensile strength and elongation.

Figure 9 and Figure 10 also illustrate the fracture surface morphologies of all MMT-added PVOH nanocomposites at various loading levels of cuttlebone. For all MMT-calcined cuttlebone-PVOH composites at fixed 1 phr MMT, the surface became rough with the increment of calcined cuttlebone, as shown in Figure 9a and Figure 10a. Tearing matrix and also fibrils were present in the composites. The agglomeration of calcined cuttlebone could not be counterbalanced by the addition of MMT in the PVOH matrix. Thus, as the amount of calcined cuttlebone increased up to 5 phr, agglomeration started to occur where the matrix showed discontinuity. Calcined cuttlebone formed hydrogen bonds within themselves rather than with the PVOH matrix. Furthermore, calcined cuttlebone tended to have phase separation, leading to poor distribution of calcined cuttlebone in the PVOH matrix. This reduction in matrix continuity resulted in brittleness of the composites. The blend would break faster when stress was applied, further decreasing tensile strength and elongation. On the other hand, the beneficial effect of the cuttlebone was only limited to certain loading levels. This could be seen when the amount of calcined cuttlebone was increased to 5 phr with fixed 2 phr and 3 phr MMT in the PVOH matrix; the tensile strength also increased as a result due to its good reinforcing effect. Less flake-like structure could also be observed compared to the 2 phr calcined cuttlebone filled composite.

## 4. Conclusions

This study was focused on the investigation of PVOH blended with calcined cuttlebone and MMT. To reiterate, when 5 phr of calcined cuttlebone was added to PVOH-MMT blends at fixed 1 phr MMT, the poor distribution of high amounts of calcined cuttlebone particles reduced the tensile strength of the composites. Stress concentration spots were formed, subsequently weakening the functionality of the physical structure. When the loading level of calcined cuttlebone increased, Young’s modulus of all composites showed a larger value, which is probably attributed to the high rigidity and stiffness of cuttlebone due to the strong intermolecular and intramolecular hydrogen bonding within the nanocomposites. In addition, the elongation of composites with fixed loading of MMT was decreased when subjected to the higher 5 phr of calcined cuttlebone loading. This is because the aggregation of calcined cuttlebone particles contributed to the formation of a stress concentration region, thus resulting in the reduction of chain sliding ability. The effects of calcined cuttlebone and MMT in composites could be observed via SEM micrographs of the fracture surfaces. The morphology of PVOH-cuttlebone-MMT composites with higher loading level of cuttlebone showed more tearing matrix and fibrils than lower loading level of calcined cuttlebone at fixed 1 phr MMT. Agglomeration was observed where the matrix showed discontinuity, causing the brittleness of composites. On the other hand, when MMT concentration was higher, at 2 phr and 3 phr, the intercalation effect of calcined cuttlebone was visible. Short fibril and less flake-like structure could be observed in 5 phr calcined cuttlebone compared to 2 phr due to the improved dispersion of calcined cuttlebone and MMT in composites, contributing to the enhanced tensile strength and poor elongation. XRD analysis confirmed that overall, the calcined cuttlebone was well dispersed until the crystallite structure disappeared. PVOH disturbed the highly ordered structure of cuttlebone, causing the disappearance of the deflection peak. The d-spacing also decreased due to low interfacial adhesion of cuttlebone, which is in agreement with the tensile strength test. The addition of calcined cuttlebone in pure PVOH showed a red shift effect, implying the formation of hydrogen bonding among the components. For FTIR analysis, it was also found that the wavenumber of O-H stretching for the composites with all fixed amounts of MMT decreased gradually with the increment of calcined cuttlebone. The calcined cuttlebone promoted the arrangement in the PVOH matrix and induced the formation of hydrogen bonds to provide the additional reinforcing interaction between components in the blends. When investigation expanded to C-H and O-H stretching, it could be noticed that the wavenumber decreased and then increased in relation to the increasing amount of MMT, which was in agreement with the tensile strength test. Moreover by comparing PVOH-MMT added with 2 and 5 phr of cuttlebone at fixed 2 phr MMT, the melting temperature increased. This is because the high concentration of cuttlebone and MMT were distributed evenly in the PVOH matrix, forming strong hydrogen bonding. In conclusion, the interaction of calcined cuttlebone and MMT in the PVOH system remains promising when the MMT and calcined cuttlebone are added at the amount of 2 phr and 5 phr, respectively. As mentioned earlier, such a triple combination has potential application as bio-compatibility material for medical applications with its inertness and physically strong characteristics.

## Figures and Tables

**Table 1:** Melting temperature, onset, and end temperature of PVOH-calcined cuttlebone-MMT composites.

Loading Level of MMT (phr)	Loading Level of Calcined Cuttlebone (phr)	Melting Temperature (°C)	Onset Temperature (°C)	End Temperature (°C)
1	2	228.52	216.77	231.71
2	2	227.94	213.23	234.22
3	2	228.90	216.44	235.01
1	5	228.36	222.65	234.38
2	5	228.64	217.77	234.57
3	5	228.47	216.75	234.60

**Table 2:** Wavenumbers of its stretching type for all PVOH-calcined cuttlebone-MMT composites.

Loading Level of MMT (phr)	Loading Level of Calcined Cuttlebone (phr)	Wavenumber (cm^−1^)		
		O-H Stretching	C-H Stretching	C-O Stretching
1	2	3260.79	2908.67	1084.52
2	2	3262.55	2938.45	1085.55
3	2	3262.08	2915.67	1045.98
1	5	3254.23	2939.87	1087.66
2	5	3258.88	2916.46	1081.41
3	5	3258.81	2940.05	1088.10

**Table 3:** d-Spacing and crystallite size at 2θ = 19.4° for all PVOH-calcined cuttlebone-MMT composite.

Loading Level of Calcined Cuttlebone (phr)	Loading Level of MMT (phr)	d-Spacing, d (Å)	Crystallite Size, L (Å)
2	1	4.58116	611.41
2	2	4.56907	84.86
2	3	4.46249	611.05
5	1	-	-
5	2	-	-
5	3	4.50829	41.61

## Data Availability

Not applicable.

## Abbreviations

MMT, montmorillonite; PVOH, polyvinyl alcohol; SDG, sustainability development goal, XRD, X-ray diffraction analysis; FTIR, Fourier transform infrared; SEM, scanning electron microscopy; CNT, carbon nanotube; SF-SWCNT, surface-free single walled carbon nanotube; DC, direct current; phr, part per hundred resin; DSC, differential scanning calorimetry; OH, hydroxyl.
